# 自体积液癌细胞培养及药敏试验对晚期肺腺癌化疗的临床应用价值

**DOI:** 10.3779/j.issn.1009-3419.2016.09.04

**Published:** 2016-09-20

**Authors:** 良 陈, 顺芳 杨, 锦琪 蒋, 颖 张, 辉 冯, 杰 曹, 歆悦 葛, 文晖 谢

**Affiliations:** 1 200030 上海，上海交通大学附属胸科医院急诊科 Department of Emergency, Shanghai Chest Hospital, Shanghai Jiaotong University, Shanghai 200030, China; 2 200030 上海，上海交通大学附属胸科医院核医学科 Department of Nuclear Medicine, Shanghai Chest Hospital, Shanghai Jiaotong University, Shanghai 200030, China; 3 200030 上海，上海交通大学附属胸科医院检验科 Department of Laboratory, Shanghai Chest Hospital, Shanghai Jiaotong University, Shanghai 200030, China

**Keywords:** 肺肿瘤, 恶性积液, 无外源性培养, 细胞, 体外化疗敏感性试验, 化疗, Lung neoplasms, Malignant effusions, Xeno-free culture, Cell, *Ex vivo* chemo-sensitivity assay, Chemotherapy

## Abstract

**背景与目的:**

晚期肺癌的化疗效果存在极大的个体差异，如何选择最佳化疗方案，实现肺癌化疗的个体化、效果最大化是值得探索的课题。本研究旨在检验自体积液中进行癌细胞培养及药敏试验对于合并胸腔或心包积液的肺腺癌患者优化化疗方案的临床应用价值。

**方法:**

收集50例并发恶性胸腔和/或心包积液的肺腺癌初治患者，经闭式引流控制积液。其中25例（药敏组）于无菌条件下留取积液300 mL-500 mL，肿瘤细胞通过自体积液（xeno-free）平均11天的细胞培养而获得，继而针对8种临床常用化疗药物行药敏试验，通过CCK-8进行敏感性检测，根据试验结果选择最优化疗方案进行全身化疗；另25例（对照组）进行经验性化疗。

**结果:**

4个周期化疗后，药敏组部分缓解（partial response, PR）17例（68.0%）、稳定（stable disease, SD）5例（20.0%），客观缓解率（objective response rate, ORR）68.0%，疾病控制率（disease control rate, DCR）88.0%；对照组PR 9例（36.0%）、SD 7例（28.0%），ORR为36.0%，DCR为64.0%。比较两组ORR和DCR，差异均有统计学意义（*P* < 0.05）。至随访截止，药敏组死亡21例，对照组死亡22例。药敏组无进展生存期（progression-free survival, PFS）平均10.0个月，总生存期（overall survival, OS）平均30.2个月；对照组PFS平均5.8个月，OS平均21.2个月。比较两组PFS和OS，差异均有统计学意义（*P* < 0.05）。两组毒副反应轻微、可控。

**结论:**

自体积液癌细胞培养及药敏试验有利于晚期肺腺癌患者化疗方案的合理选择。

## Abstract (en)

**Background and objective:**

A great individual differences to chemotherapeutic effects existed in the patient with advanced lung cancer. How to choose the optimum regimens to achieve the individuation and maximum effect of chemotherapy for lung cancer is worth exploring. The study was designed to examine the effect of ex vitro chemo-sensitivity assay in xeno-free culture of autologous malignant effusion cells from patients with advanced lung adenocarcinoma.

**Methods:**

The 50 treatment-naive patients with lung adenocarcinoma complicated with malignant pleural or pericardial effusions were enrolled. Effusions of all cases had been controlled by closed drainage and 300 mL-500 mL of which were retained under sterile condition from 25 cases (Chemo-sensitivity group). Primary malignant effusion cells were isolated from autologous effusions of the patients. Then, xeno-free culture (average 11 days) were intervened with 8 chemotherapeutic drugs commonly used in clinical practice and were determined by CCK-8 assay. Optimum regimens were selected for chemotherapy based on the results of chemosensitivity test. As a contrast, chemotherapy regimens for the other 25 patients (Control group) were on the basis of physician's clinical experience.

**Results:**

After four cycles of chemotherapy, in Chemo-sensitivity group, 17 (68.0%) cases were determined for partial response (PR), 5 (20.0%) cases for stable disease (SD), and the objective response rate (ORR) was 68.0%, the disease control rate (DCR) was 88.0%. Meanwhile, in Control group, 9 (36.0%) cases were determined for PR, 7 (28.0%) cases for SD, and, the ORR was 36.0%, the DCR was 64.0%. There were significant differences between the two groups in ORR and DCR (*P* < 0.05). To the end of follow-up, there were 21 cases of death in Chemo-sensitivity group as well as 22 cases in Control group. The mean progression-free survival (PFS) in Chemo-sensitivity group and Control group respectively were 10.0 months and 5.8 months, and the mean overall survival (OS) in the two groups were 30.2 months and 21.2 months respectively. There were also significant differences between the two groups in PFS and OS (*P* < 0.05). Furthermore, the adverse reactions in both groups were mild and controllable.

**Conclusion:**

Xeno-free culture of autologous malignant effusion cells from patients with advanced lung adenocarcinoma and *ex vitro* chemo-sensitivity assay are beneficial to the rational choices of chemotherapeutic agents used in patients with lung adenocarcinoma complicated with malignant effusions, which is a worthy trial in personalized cell culture for individualized cancer therapy and further studies.

随着肺癌研究的不断深入，针对肺癌的新技术、新药物的运用取得了长足进步，然而依然没有一项新技术、一种新药物能完全取代化学治疗，化疗依然是中晚期肺癌的常规治疗手段。然而，肺癌的异质性、多态性、耐药性必然要求治疗的个体性，因此如何选择最佳化疗方案、实现肺癌化疗的个体化以及效果的最大化仍是临床值得进一步探索的课题。本研究尝试从晚期肺腺癌患者的胸腔或心包积液提取癌细胞，在自体积液中培养并进行化疗敏感性试验（简称药敏试验），以指导化疗方案的选择，通过临床化疗效果评判该方法对晚期肺腺癌化疗的指导价值。

## 1 资料与方法

### 1.1 临床资料

收集我科2010年4月-2013年3月收治的肺癌患者50例（表 1），其中男性24例，女性26例，年龄54岁-66岁。结合影像学和病理学确诊为原发性肺腺癌，均合并胸腔积液和/或心包积液，积液细胞学检查明确腺癌细胞的存在，其中部分患者同时合并骨、脑、肺等远处脏器的转移，按国际抗癌联盟（Union for International Cancer Control, UICC）第7版分期^[1]^均为Ⅳ期病例。所有患者都具有影像学可评估的靶病灶，且其主要脏器功能正常，积液引流后全身情况良好，功能状态评分（Zubrod-ECOG-WHO score, ZPS）≤2分，无化疗禁忌症，均能耐受全身化疗，且既往无放疗和/或化疗病史。所有患者在整个生命期内均无靶向药物应用史。随访截止日期至2016年3月31日。

**1:** 50例肺腺癌患者的临床特征、治疗效果和生存时间 Clinical characteristics, therapeutic effects and survival time of 50 patients with lung adenocarcinoma

Parameter	CSG (*n*=25)	CG (*n*=25)	*P*
Gender [*n* (%)]			
Male	12 (48.0)	12 (48.0)	
Female	13 (52.0)	13 (52.0)	
Mean age (yr, Mean±SD)	59.4±4.4	61.1±3.2	
Metastases [*n* (%)]			
Bone	6 (24.0)	8 (32.0)	
Brain	4 (16.0)	3 (12.0)	
Lung	5 (20.0)	4 (16.0)	
Effusion location [n (%)]			
Pleural	12 (48.0)	13 (52.0)	
Pericardial	5 (20.0)	6 (24.0)	
Pleural & Pericardial	8 (32.0)	6 (24.0)	
Zubrod-ECOG-WHO score [*n* (%)]			
1	13 (52.0)	12 (48.0)	
2	12 (48.0)	13 (52.0)	
Therapeutic effect [*n* (%)]			
CR	0 (0)	0 (0)	
PR	17 (68.0)	9 (36.0)	
SD	5 (20.0)	7 (28.0)	
PD	3 (12.0)	9 (36.0)	
ORR	17 (68.0)	9 (36.0)	0.023, 5
DCR	22 (88.0)	16 (64.0)	0.046, 9
Survival time (mo, Mean±SD)			
PFS	10.0±1.3	5.8±1.1	0.012, 5
OS	30.2±2.8	21.2±2.1	0.023, 4
CSG: chemo-sensitivity group; CG: control group; CR: complete response; PR: partial response; SD: stable disease; PD: progressive disease; ORR: objective response rate; DCR: disease control rate; PFS: progression-free survival; OS: overall survival.			

### 1.2 病例分组

所有病例在治疗前均通过胸腔/心包闭式引流术将积液完全引尽，胸腔内注入沙培林（注射用A群链球菌）行胸膜粘连术，心包内注入卡铂行局部化疗。积液控制后10 d-14 d进行全身化疗。

入选患者中有40例在胸腔/心包积液引流时于无菌条件下留取积液300 mL-500 mL，分离肿瘤细胞后于自体（xeno-free）积液中进行细胞培养。其中25例（62.5%）成功获取肿瘤细胞并能培养扩增至目标数量（见下文1.3节），入选药敏组（chemo-sensitivity group, CSG）进行药敏试验，并根据试验结果选择最佳化疗方案进行全身化疗；其余15例未能有效培养出目标数量肿瘤细胞以及10例未行细胞培养的患者列为对照组（control group, CG），直接进行经验性化疗。

### 1.3 癌细胞培养及药敏试验方法

参照以往方法^[2-5]^，将采集的新鲜积液加肝素钠（10 U/mL）抗凝，并加入青霉素（100 U/mL）、链霉素（100 μg/mL）和庆大霉素（5 U/mL）抗菌，随机将部分积液移入10个25 cm^2^细胞培养瓶，于37 ℃、5%CO_2_德国贺利氏BB16二氧化碳孵养箱内孵育。剩余积液作为培养基于孵箱内保存备用。24 h后，取培养瓶、弃上清液、保留瓶底贴壁细胞，加入备用积液的上清液平均培养11 d，见贴壁细胞总数在30×10^4^-40×10^4^，取培养瓶、弃上清，用细胞刮将细胞刮下，加入含10%胎牛血清的RPMI-1640培养液（Gibco, Carlsbad），以吸管吹打后移入15 mL离心管离心，弃上清，加入RPMI-1640培养液12 mL，吹打均匀后按3×10^3^-5×10^3^个细胞/0.1 mL/孔接种于96孔细胞培养板^[6]^。

孵育24 h后，按各化疗药物的人体峰药浓度设高、中、低浓度药物组（以峰药浓度为高浓度组，依次稀释10倍为中、低浓度组）和癌细胞对照组，每组3复孔。所用化疗药物选用目前临床常用方案，包括培美曲塞（pemetrexed, PEM）、吉西他滨（gemcitabine, GEM）、长春瑞滨（vinorelbine, NVB）、多西他赛（docetaxel, DOC）、紫杉醇（taxol, TXL）、异环磷酰胺（ifosfamide, IFO）、顺铂（cisplatin, DDP）和卡铂（carboplatin, CBP）。加药设组后继续培养72 h，调整液体体积为0.1 mL/孔，加入CCK-8反应液（Dojindo, Kumamoto）0.01 mL/孔。孵育1 h后以酶标仪450 nm波长检测吸光度（optical density, OD）值^[7]^，药物组OD值取3复孔平均值，计算癌细胞相对抑制率（inhibition rate, IR）=（对照组OD值-药物组OD值）/对照组OD值×100%^[8]^。IR > 30%判为敏感，70% > IR≥50%为中度敏感，IR≥70%为高度敏感。

### 1.4 化疗方案及疗效评价

所有患者均采用以铂类为基础的标准二联化疗方案，每2个周期评判化疗效果，但为了便于统计学分析，前4周期化疗无论效果如何均保持方案不变（化疗前均需签署知情同意书）。4个周期后，疗效判为有效者继续巩固化疗，至疾病进展时按经验选择二线乃至三线方案化疗；疗效稳定者共化疗6个周期后进行随访观察；而疗效判为进展者则直接按经验选择二线乃至三线方案化疗。当化疗的临床获益消失或患者无法继续耐受化疗时则终止化疗而随访。其中，药敏组患者以药敏试验中癌细胞相对抑制率最高者为优选标准，PEM、GEM、NVB、DOC、TXL和IFO中取其一，DDP和CBP取其一；对照组以经验方案化疗。

疗效评判标准包括：客观缓解率（objective response rate, ORR）和疾病控制率（disease control rate, DCR）：以4个周期化疗后胸部X线计算机体层摄影（computed tomography, CT）检查为参考，按2009年实体瘤疗效评估标准（Response Evaluation Criteria in Solid Tumors, RECIST）1.1版^[9]^对照靶病灶变化，根据病灶的最大长径的总和计算，判为：①完全缓解（complete response, CR）：所有靶病灶全部消失；②部分缓解（partial response, PR）：所有靶病灶的最大长径总和减少30%以上；③病变进展（progressive disease, PD）：观察期间与最小值相比最大长径的总和增加20%以上或出现新病灶；④稳定（stable disease, SD）：既不能满足PR又不能满足PD的病变。ORR=CR+PR；DCR=CR+PR+SD。无进展生存期（progression-free survival, PFS）：从治疗开始至肿瘤发生恶化或死亡的时间。总生存期（overall survival, OS）：治疗开始至死亡或末次随访的时间。

### 1.5 统计学方法

采用SAS 8.1软件包进行统计学处理。两组治疗总缓解率和疾病控制率的比较采用χ^2^检验，生存分析采用*Kaplan*-*Meier*法，生存期比较采用*Log*-*rank*检验，均以*P* < 0.05为差异有统计学意义。

## 2 结果

### 2.1 癌细胞培养及药敏试验结果

药敏组患者的原代腺癌细胞于自体积液中获得良好生长和扩增（图 1示其中一例自体胸腔积液培养的原代肺腺癌细胞），符合药敏试验要求。

**1:**
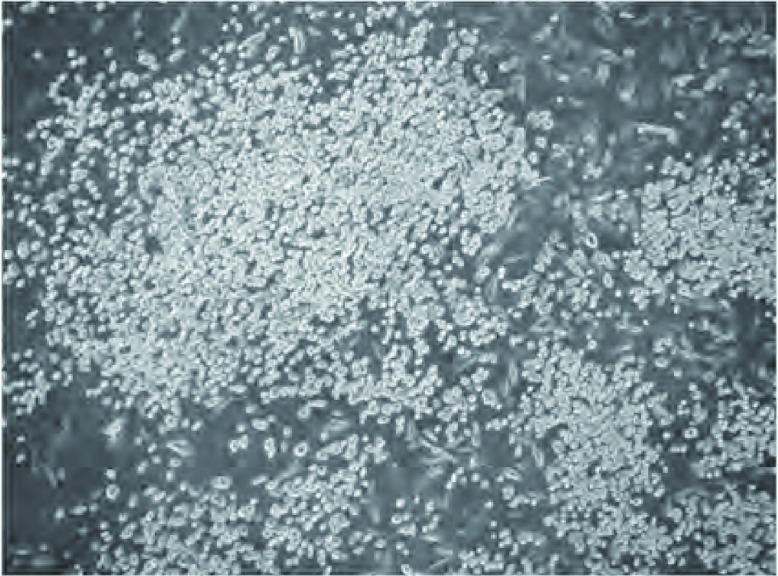
一例57岁女性肺腺癌患者自体胸腔积液培养7 d的癌细胞相差显微镜图（×40） The autologous carcinomatous cells derived from malignant pleural effusion in a 57 yr female with late-stage lung adenocarcinoma under the contrast phase microsope (×40)

所用化疗药物在体外对自体积液培养的癌细胞的抑制率见表 2。各药物的平均抑制率随药物浓度的增加而增高；除中、低浓度的PEM对细胞的平均抑制率低于30%，其余药物在不同浓度下的抑制率均 > 30%；在中浓度时，除了PEM和GEM，其余药物的抑制率均 > 50%；而在高浓度时，除了PEM，其余药物的抑制率均 > 50%；所有药物的平均抑制率均未超过70%。

**2:** 各化疗药物对肺腺癌细胞的抑制率（%, Mean±SD） Inhibitory rates of chemotherapy drugs for lung adenocarcinoma cells (%, Mean±SD)

Chemotherapy drugs	High concentration	Middle concentration	Low concentration
PEM	47.2±20.7	29.4±22.9	26.6±23.7
GEM	57.9±20.9	49.5±24.1	35.1±23.1
NVB	63.7±17.2	53.1±15.1	44.1±22.6
DOC	67.2±16.4	61.5±16.3	49.7±24.6
TXL	65.7±16.5	54.3±17.4	34.9±24.1
IFO	69.2±17.6	45.2±29.5	31.4±29.5
DDP	67.3±13.6	56.3±15.9	42.1±24.2
CBP	68.8±12.8	52.4±23.6	31.1±23.8
PEM: pemetrexed; GEM: gemcitabine; NVB: vinorelbine; DOC: docetaxel; TXL: taxol; IFO: ifosfamide; DDP: cisplatin; CBP: carboplatin.			

在非铂类药物中，对平均抑制率由高到低排列，低浓度时依次为DOC、NVB、GEM、TXL、IFO和PEM，中浓度时依次为DOC、TXL、NVB、GEM、IFO和PEM，而高浓度时则为IFO、DOC、TXL、NVB、GEM和PEM；铂类中，中、低浓度时DDP的平均抑制率略高于CBP，而高浓度时DDP与CBP相仿。然而就单个病例，各药物的抑制率高低与总人群的平均抑制率的排序并非完全一致。

### 2.2 化疗效果及生存情况分析

所有50例患者均顺利完成4个以上周期的全身化疗，4个周期化疗后的临床疗效及所有患者的生存情况见表 1。两组患者中均无CR病例，药敏组25例患者中PR 17例（68.0%）、SD 5例（20.0%），ORR 68.0%，DCR 88.0%；对照组PR 9例（36.0%）、SD 7例（28.0%），ORR 36.0%，DCR 64.0%。经统计学χ^2^检验，分别进行两组ORR和DCR比较，差异有统计学意义（*P* < 0.05）。

至随访截止，药敏组死亡21例，对照组死亡22例，均为肿瘤相关性死亡。对两组患者生存情况进行*Kaplan*-*Meier*法分析，药敏组PFS为0个月-24个月，平均10.0个月，OS为6.1个月-71.2个月，平均30.2个月；对照组PFS为0个月-21个月，平均5.8个月，OS为5.8个月-44.8个月，平均21.2个月。经*Log*-*rank*检验，分别比较两组PFS和OS，差异均有统计学意义（*P* < 0.05）（图 2）。

**2:**
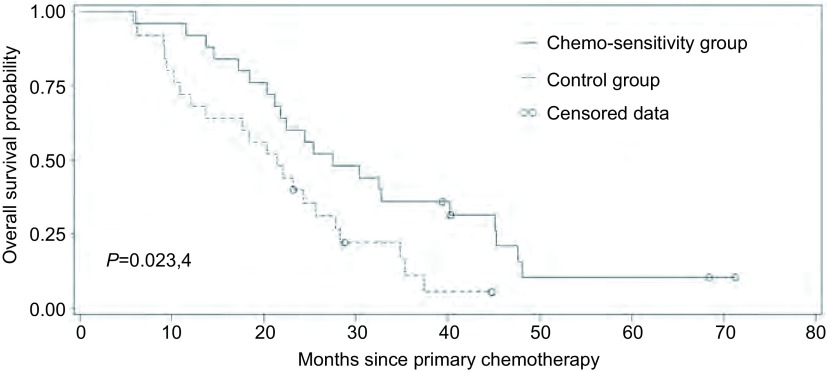
两组患者总生存期曲线（*Kaplan*-*Meier*），差异有统计学意义（*P*=0.023, 4） *Kaplan*-*Meier* curves of overall survival probabilities for patients with advanced lung adenocarcinoma, there was significant difference between the two groups (*P*=0.023, 4).

### 2.3 化疗毒副反应

两组患者化疗期间常见毒副反应包括乏力、食欲减退、呕吐、外周血白细胞下降、贫血、血小板减低、静脉炎等，其发生率及严重程度因所用药物不同而有所差异，经对症治疗后患者均能较好耐受，无一例因毒副反应而终止化疗或调整化疗剂量。

## 3 讨论

时至今日，中晚期肺癌尤其合并胸腔或心包积液患者的预后依然不佳。虽然分子靶向药物的研发和应用为非小细胞肺癌（non-small cell lung cancer, NSCLC）的预后、生存质量的改善作出了里程碑式的贡献，然而由于患者个体差异的存在、基因突变位点的不同，以及当前对于肿瘤相关突变基因的检测能力有限而无法完全获取所有的靶点信息，导致靶向药物的获益人群和获益时间均十分有限。因此，虽然化疗的效果有限且存在一定的毒副作用，但其依然是现阶段多数中晚期肺癌患者延长生命时间的重要手段。

目前针对NSCLC化疗方案的选择较大依赖于临床医师的治疗经验，缺乏个体化选择标准，因此不可避免存在一定的盲目性，故而无法实现肿瘤化疗的最佳效益，导致化疗“瓶颈”的出现。而癌细胞药敏试验为化疗方案的选择开辟了新的方向。Iwahashi等^[10]^报道了64例行胃大部切除术的胃癌患者在药敏试验指导下进行全身化疗后的5年生存率比经验化疗有明显提高。Lau等^[11]^发现在药敏试验指导下化疗的乳腺癌患者比经验性化疗者得到了更高的临床缓解率（10/24 *vs* 0/12），肿瘤面积的缩小率亦有提高（75% *vs* 26%）。国内聂磊等^[12]^也通过药敏试验结果指导24例肝细胞癌患者的肝动脉和门静脉区域化疗，与对照组相比，缓解率（50.0% *vs* 20.0%）、疾病控制率（70.8% *vs* 35.0%）及疾病进展时间[（4.7 ±2.9）个月*vs*（2.6±1.3）个月]均有明显优势。而对于肺癌，Kawamura等^[13]^曾通过胶滴肿瘤药敏检测技术对49例NSCLC患者行药敏试验，其中22例存在敏感药物而进行了药敏试验指导下的全身化疗，其临床缓解率为72.7%、中位生存期为15个月；而另11例试验不敏感者进行了经验性化疗，其缓解率为0、中位生存期仅为6个月。

然而，现有研究中药敏试验所采用的肿瘤细胞几乎均来源于手术切取或细针穿刺所得的肿瘤组织，通过酶解或钢网滤过等方法而获得，在胎牛血清等特定培养液中培养，继而采用四氮唑蓝（MTT）、ATP生物荧光法（ATP-TCA）等^[14]^方法进行抗癌药物敏感性测定。由于肿瘤细胞来源于实体组织块，离体后却在非自身来源的培养液中生长，其离体和在体生长环境、营养来源存在极大差异，导致肿瘤离体后的生长方式、生物学特性发生了一定程度的改变，离体细胞对药物的敏感性与体内肿瘤对化疗的反应性并非完全一致，从而丧失了药敏试验指导临床化疗的优越性。Wu等^[8]^通过对353例胃癌患者进行对照性研究，其中157例按MTT法药敏试验结果指导化疗、196例按经验方案化疗，最终发现药敏组的5年生存率为47.5%、经验组为45.1%，差异无统计学意义，可能就有以上原因存在。

晚期肺癌易发生胸膜腔或心包腔转移而导致胸腔和/或心包积液的生成，而胸腔/心包积液正是肺癌细胞的最好来源，其获取途径安全、便利。同时积液又是肺癌细胞的天然培养基，癌细胞离体后在自体积液环境中培养能最大程度的模拟体内环境，从而保持与体内相同的生长方式和生物学特性以及对药物的敏感性。因此，胸腔/心包积液来源并于自体积液培养下的癌细胞药敏试验结果在一定程度上能更好地反映在体肿瘤对化疗药物的敏感性，从而能更好地指导临床化疗方案的选择和患者的个体化治疗，提高治疗效果，最终使中晚期肺癌患者能更多的从化疗中获益。

本研究以肺腺癌患者为研究对象，是因为其胸腔/心包积液中较其他类型肺癌更容易获取转移性癌细胞，从而提高细胞培养及药敏试验的成功率。通过研究发现，药敏试验指导下全身化疗的ORR和DCR分别为68.0%和88.0%，明显优于对照组的36.0%和64.0%；进而，药敏组的PFS和OS平均分别为10.0个月和30.2个月，亦明显长于对照组5.8个月和21.2个月。且两组化疗的毒副反应均轻微、可控，其发生率及严重程度主要与不同药物的自身特性相关。因此，自体积液癌细胞培养后的药敏试验对晚期肺腺癌化疗方案的选择具有一定的临床应用价值。

然而，通过试验结果发现，目前常用化疗药物对肺癌细胞的体外抑制率总体不高，平均抑制率均未超过70%，从而可以解释药敏组患者全身化疗的效果虽然优于对照组但总体疗效依然并不理想的原因。因此，期待更大样本对照研究的开展和更安全有效抗肿瘤药物的研发以进一步提高中晚期肺癌患者个体化治疗的效果，实现最大限度地延长生命时间、提高生命质量。
